# External validation of the Liverpool Head Injury Tomography Score for mild traumatic brain injury

**DOI:** 10.1007/s00701-026-06991-4

**Published:** 2026-08-04

**Authors:** Conor S. Gillespie, Yuhan Guo, Matthew Kingham, Shangqing W. Yang, Zhikai Li, Munashe Veremu, William H. Cook, Youssef Chedid, Emily Simmons, Naomi Deakin, David Clark, Virginia F. J. Newcombe, Peter J. Hutchinson, Catherine J. McMahon, Alexis J. Joannides, East of England Emergency Neurosurgery Collaborative, Prabhjot Singh Malhotra, Benjamin M. Davies, Sara Venturini, Edoardo Viaroli, Mutwakil Abdulla, Adam M. H. Young, Yevgeny Karepov, Muhammad Shuaib Khan, Oliver Mowforth, Midhun Mohan, Saeed Kayhanian, Richard Bartlett, Stefan Yordanov, Agnieszka Zakrzewska, Francisca Ferreira, Kirsty Grieve

**Affiliations:** 1 Department of Neurosurgery, Addenbrooke’s Hospital, Cambridge, UK; 2 Department of Clinical Neurosciences, University of Cambridge, Downing Street, Cambridge, CB2 3EB UK; 3 Orion MedTech Ltd. CIC, Cambridge, UK; 4 Perioperative, Acute, Critical, and Emergency Care (PACE) Section, Department of Medicine, University of Cambridge, Cambridge, UK; 5 Department of Neurosurgery, Salford Royal NHS Foundation Trust, Manchester, UK; 6 NIHR HealthTech Research Centre in Brain Injury, Cambridge University Hospitals NHS Foundation Trust, Cambridge, UK

**Keywords:** TBI, Head injury, Mild TBI, Neurosurgery, Referral

## Abstract

**Background:**

Mild Traumatic Brain Injury (mTBI) neurosurgical referrals are increasing. This study aimed to externally validate a radiological scoring system to define a ‘neurosurgically significant’ mild TBI and evaluate its impact on referral outcome if implemented.

**Method:**

Retrospective, external validation of the UK Liverpool Head Injury Tomography Score (LIV-HITS). Consecutive neurosurgery referrals to a tertiary neurosurgery centre for mild TBI were included (1st January 2022—30th June 2022). Mechanism of injury, LIV-HITS and management decision were identified, and diagnostic accuracy determined.

**Results:**

A total of 1547 referrals were included. 147 (9.5%) were accepted for transfer, and 1400 (90.5%) were designated as local management. For neurosurgical transfer, the sensitivity of LIV-HITS was 99.3% and the specificity was 43.9%, with a positive predictive value of 15.7%, and negative predictive value of 99.8%. Discriminatory power of the model was fair (AUC 0.71, 95% CI 0.66–0.76). In 6 months, the score identified 615 referrals (39.8%) that could have been successfully triaged to local management if the scoring system was used.

**Conclusions:**

The LIV-HITS has a sensitivity of over 99% for excluding a neurosurgically significant mild TBI in an externally validated cohort. However, the discriminatory power of the score is poor for stratifying which patients eventually get transferred to a neurosurgical centre. The score in clinical practice could be used as a ‘rule out’ tool for mild TBI referrals, whilst identifying potentially significant mild TBI that would warrant emergency neurosurgical consideration.

## Introduction

Traumatic brain injury (TBI) remains a worldwide public health concern, with an estimated 64–74 million people affected per year [10]. Patients with TBI can be broadly classified according to their Glasgow Coma Scale (GCS) score on presentation, into mild (GCS: 13–15), moderate (GCS: 9–12), and severe (GCS: 3–8) [19, 34]. In clinical practice, mild TBI (mTBI) constitutes the majority (over 90%) [19] of TBI cases presenting to hospital. It therefore represents a significant proportion of referrals taken by the on-call neurosurgeon, whose role is often to decide whether patients with mild TBI in local hospitals require emergency neurosurgical advice, or management.

In recent years, the incidence of TBI presentations to the emergency department (ED) appears to be increasing, with some studies reporting more than a 50% rise in the volume of cases [12, 27]. Despite this, evidence exists suggesting that the overall acceptance rates, indicating surgical management, to neurosurgical units from local hospitals remain low [9] in the United Kingdom. Additionally, it is well documented that on-call neurosurgeons receive a high volume of referrals [16, 22], many of which are urgent or emergent cases that require rapid decision making. A safe way of stratifying mild TBI referrals may therefore provide a rapid method of triaging patients to the appropriate referral stream, in addition to alleviating the practical and financial burden on receiving units [13, 16, 30]. In hospitals without neurosurgical facilities, it can be difficult to establish whether a mild TBI is ‘surgically significant’ – a referral that requires either conservative or operative management in a specialist neurosurgical unit. The issue can be further complicated by medicolegal pressure [23], and therefore a clear, documented rationale to justify managing a head injured patient locally is essential.

**Fig. 1:**
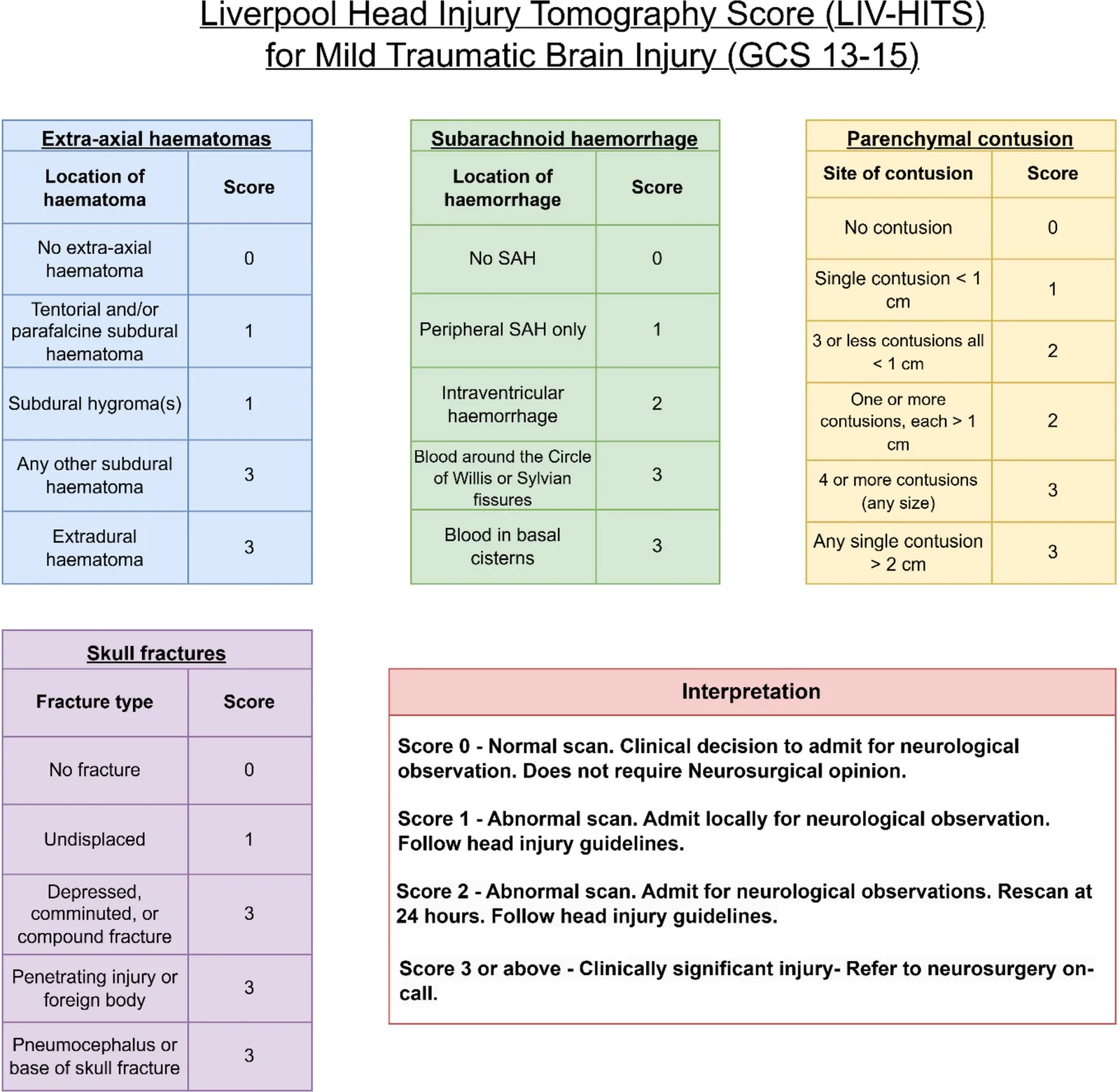
Liverpool Head Injury Tomography Score (LIV-HITS)

The Liverpool Head Injury Tomography Score (LIV-HITS) (Fig. 1) is a four-point scoring system that was developed and validated on a retrospective sample of 1144 patients with mild TBI, referred to the on-call neurosurgery department at a tertiary referral centre [9]. The tool was developed as a ‘rule out’ tool for mild TBI, with secondary aims of improving time to advice and neurosurgical input for local units. It is comprised of four different parameters—extra-axial haematoma(s), (extent of) subarachnoid haemorrhage, parenchymal contusion(s) and skull fracture(s). An overall score is calculated, with a score of ‘3’ or more determined to be a neurosurgically-significant injury- one that requires immediate discussion with an on-call neurosurgeon for advice, and potential transfer to a specialist centre offering neurosurgical services. In the original study, the score was found to be 99.0% sensitive and 51.9% specific on internal validation of a mild TBI cohort, with an area under the curve (AUC) of 0.791 (95% CI: 0.76–0.82) [9], reflecting the general trend of low overall transfer rates from district general hospitals to specialist neurosurgical centres [30].

Previous NICE guidance CG176 (2014) [24] recommended discussing all ‘surgically significant’ traumatic brain injuries with a neurosurgeon, and that the definition of ‘surgically significant’ should be locally determined. Updated 2023 guidance (NG232) suggests discussing all patients with ‘new and surgically significant abnormalities on imaging’, with the definition of ‘surgically significant’ agreed locally by the local neurosurgical centre and referring institution, introducing subjectivity to deciding whether to refer.

The LIV-HITS thus provides an objective metric to define a ‘surgically significant’ injury or abnormality requiring neurosurgical consultation, and possible management. The score, however, requires external validation to establish reproducibility in different patient populations [6, 26]. The aim of this study was therefore to externally validate and assess the predictive performance of LIV-HITS in a cohort of mild TBI referrals to a separate neurosurgery referral centre, using acceptance for transfer versus local management as the referral outcome.

## Methods and materials

### Study design, setting and participants

This study was a single-centre, retrospective external validation of LIV-HITS on a consecutive series of 1547 patients over the age of 16 years, referred to a single tertiary neurosurgery centre with mild TBI (GCS 13–15), over a period of 6 months (01/01/2022–30/06/2022). We chose a period of 6 months to capture an appropriately large number of referrals for external validation. Referrals were from eleven local hospitals within the East of England Trauma Network, a catchment area of over 6 million people. All mild TBI referrals within the study period were included in the analysis. There was no overlap in either individual patient data or the referral hospitals when comparing the development and validation cohorts. Additionally, although LIV-HITS is now used in the centre in which it was developed, it was not in use during the study period nor in the referral process of the validation centre. The study was approved locally by the hospital’s audit department as a service evaluation project. Audit approval from the hospital trust was obtained before commencing the study (Clinical Project ID: 6492). As a service evaluation, individual patient consent was not required.

### Data extraction and referral characteristics

The dataset was extracted from ORION, an online neurosurgical referral system used in both the development and validation neurosurgical centres (without patient data overlap). ORION is an electronic neurosurgical referrals system whereby referrers from secondary hospitals refer for neurosurgical consultation and advice (https://orion.net/) [14]. Referral data submitted includes patient identifiers, history, medical history, current medication, and examination findings including GCS, neurological examination, and pupil reactivity, in addition to scan results and radiological reports. The outcome of the referral is then communicated electronically back to the referrer (local management or transfer to neurosurgical unit), with the option of further discussion or clarification.

Each unit analysed was a single ORION referral episode. Data extracted included the date of referral, the GCS of the patient on presentation, the LIV-HITS at the time of referral, the working diagnosis and whether the patient was accepted and transferred to a specialist neurosciences centre. There were two main outcomes of referral analysed; transfer to the regional neurosurgical centre or advice for local management. Duplicate referrals were excluded, unless the referral was for a new injury or clinical question.

The primary outcomes were the sensitivity, specificity and AUC of the LIV-HITS in this patient population, as well as the gross acceptance percentage for transfer to a tertiary unit. LIV-HITS was independently assessed by two study authors (MK and CSG), and any conflicts discussed with the senior author (AJJ). We also compared the baseline demographics from the East of England cohort, compared to the Liverpool cohort (dataset obtainable from original study) [9].

### Statistical analysis

Overall referral volume and acceptance rates were presented as descriptive statistics. For validation, the scoring parameters in the original LIV-HITS publication were used; an accepted referral with a LIV-HITS of 3 or more was defined as a ‘true positive’ case; with LIV-HITS of 3 or more leading to local management defined as a false positive (Table 1). Cases where the score was less than 3 with local management were defined as a ‘true negative’, and cases that were transferred where the score was less than 3 were defined as ‘false negative’ cases. Sensitivity, specificity and positive and negative predictive values were then calculated from this classification. Estimated potential reduction in referrals was calculated from the proportion of true negative cases among all included referrals. AUC was determined using the trapezoid rule, and Receiver Operating Characteristic (ROC) curves were generated using RStudio Version 4.0.1 (pROC package), based on established methods [9]. Differences in baseline and referral characteristics between the Liverpool and East of England cohorts were analysed using Chi-squared or Student's t-test where applicable.

**Table 1:** Referral outcome classification by HITS score

Outcome	LIV-HITS ≥ 3	LIV-HITS < 3
Transferred to Neurosurgical Centre	True Positive	False Negative
Not transferred to Neurosurgical Centre	False Positive	True Negative

### Definitions

For the electronic referrals system, a neurosurgical consultation was defined as a single ORION referral episode for neurosurgical advice regarding a patient with mild TBI. A ‘surgically significant injury’ was defined as a mild TBI presentation, in which clinical or radiological features suggested that neurosurgical management (transfer to a tertiary neurosciences centre or immediate advice and guidance) was required. Such features would include: subdural haematoma (both acute and chronic), multiple contusions, complex/displaced skull fractures, and multiple injuries. The aim of defining ‘surgical significance’ as one that required neurosurgical transfer was to ensure a granular definition on which performance of the score could be measured. Admission to the centre is also pragmatic for the population served (6 million people across 12 hospitals). In the absence of neurosurgical transfer, other management streams and pathways (such as neurorehabilitation/outpatient appointment/concussion management) were still possible and accessible through appropriate referral channels.

## Results

A total of 1547 patients with mild TBI were referred during the study period. The baseline demographics, and referral outcomes are shown in Tables 2 and 3. Most patients referred were GCS 15 (73.0%, *n* = 1130/1547). The most common working diagnosis was chronic subdural haematoma (24.4%, *n* = 377/1547).

**Table 2:** Baseline demographics

**Characteristic**	***N*** (%)
Number of referrals included	1547
Mean Age (SD)	72.2 (19.7)
Over 65	1111 (71.8)
Male	853 (55.1)
**GCS**	***N*** (%)
15	1130 (73.0)
13–14	417 (27.0)
Anticoagulant/antiplatelets	311 (20.1)
**Working diagnosis**	**Value (%)**
Acute subdural haematoma	341 (22.0)
Focal brain injury	344 (22.2)
Chronic subdural haematoma	377 (24.4)
Traumatic SAH	205 (13.3)
Unspecified injury to head*	142 (9.2)
Fracture of skull vault	43 (2.8)
Base of skull fracture	33 (2.1)
Diffuse brain injury	32 (2.1)
Extradural haematoma	19 (1.2)
Other	11 (0.7)
Accepted for transfer	147 (9.5)
Local management	1400 (90.5)

*Diagnosis of head injury without intracranial abnormality present

**Table 3:** Differences between cohorts accepted for transfer and local management

Factor	Local management	Transfer
GCS 13	71 (85.5)	12 (14.5)
GCS 14	298 (89.2)	36 (10.8)
GCS 15	1031 (91.2)	99 (8.8)
Age ≥ 65	1011 (91.0)	100 (9.0)
Age < 65	389 (89.2)	47 (10.8)
Male	756 (88.6)	97 (11.4)
Female	644 (92.8)	50 (7.2)
Anticoagulant	285 (91.6)	26 (8.4)
No anticoagulant	1115 (90.2)	121 (9.8)
CSDH	282 (74.8)	95 (25.2)
ASDH	325 (95.3)	16 (4.7)
Focal brain injury	330 (95.9)	14 (4.1)
Diffuse brain injury	25 (78.1)	7 (21.9)
Extradural haematoma	13 (68.4)	6 (31.6)
Traumatic SAH	203 (99.0)	2 (1.0)
Unspecified injury to head*	141 (99.3)	1 (0.7)
Other	81 (93.1)	6 (6.9)

*Diagnosis of head injury without intracranial abnormality present

### Comparisons between East of England and Liverpool cohort

The differences between the East of England and Liverpool cohort are shown in Table 4. The mean age of patients referred to East of England was 72.2, and in Liverpool the mean age was 69.0, with a greater proportion of referrals for adults aged over 65 years in the East of England cohort (*p* < 0.05).

**Table 4:** Differences in referral characteristics between Liverpool and East of England cohorts

Characteristic	Liverpool (*n* = 1144)	East of England (*n* = 1547)
Mean age	69.0 (20.2)	72.2 (19.7)
ASDH	264 (23.1)	341 (22.0)
CSDH	200 (17.5)	377 (24.4)
Focal brain injury	182 (15.9)	344 (22.2)
Traumatic subarachnoid haemorrhage	102 (8.9)	205 (13.3)
EDH	35 (3.1)	19 (1.2)
HITS 0	198 (17.3)	99 (6.4)
HITS 1	212 (18.5)	399 (25.8)
HITS 2	85 (7.4)	118 (7.6)
HITS 3	340 (29.7)	722 (46.7)
HITS 4 or more	309 (27.0)	209 (13.5)
Transfer (%)	195 (17.0)	147 (9.5)

Chronic subdural haematomas constituted 17.5% of all referrals to the Liverpool cohort, compared to 24.4% of referrals to East of England, although the difference was not statistically significant (*p* = 0.102). In both cohorts, this diagnosis was also the most common reason for transfer, with the effect strongest in older adults at East of England (*p* < 0.001). Further analysis revealed a highly significant association between age group and diagnosis type, with older adults (age > 65 years) much more likely to reveal a diagnosis of chronic subdural haematoma than younger adults (*p* < 0.001). The frequency of acute subdural haematoma was also higher in the Liverpool cohort (23.1% vs 22.0%) as was extradural haematoma (3.1% vs 1.2%).

### Referral characteristics and external validation

Of referred patients, 9.5% (*n* = 147/1547) were accepted for transfer to the regional neuroscience unit. The remaining patients (90.5%, *n* = 1400/1547) were given advice for management and observation at their respective referring unit by the on-call neurosurgeon.

Of the patients who were not accepted for transfer, the mean LIV-HITS was 2.42 (SD: ± 1.56), with 56.1% (*n* = 785/1400) having a LIV-HITS of 3 or more, with the most common score being 3 (*n* = 605/1400, 43.2%). Of the patients who were accepted, the mean LIV-HITS was 3.49 (SD: ± 1.20). The most common score given was 3 (n = 117/147, 79.6%). Median GCS at presentation was 15 for both groups.

Referral outcome against HITS score is shown in Table 5. Of the 147 patients accepted for transfer, 99.3% (*n* = 146/147) had a LIV-HITS of 3 or above. The one patient with a LIV-HITS of less than 3 was admitted under neurosurgery with no major injuries on their CT scan (Trauma CT negative, diagnosed with concussion) due to hospital operational pressures under neurosurgery rather than clinical reasons. The patient had a concussion, was admitted for observation, and discharged after 48 hours observation. Of note, there were no data available in either dataset to establish the true safety of the transfer practice pattern and whether there were standardised protocols in place for this.

**Table 5:** Referral outcome against HITS score

Outcome	LIV-HITS ≥ 3	LIV-HITS < 3
Transferred to Neurosurgical Centre	146 (9.4%) (True Positives)	1 (0.1%) (False Negatives)
Not transferred to Neurosurgical Centre	785 (50.7%) (False Positives)	615 (39.8%) (True Negatives)

**Fig. 2:**
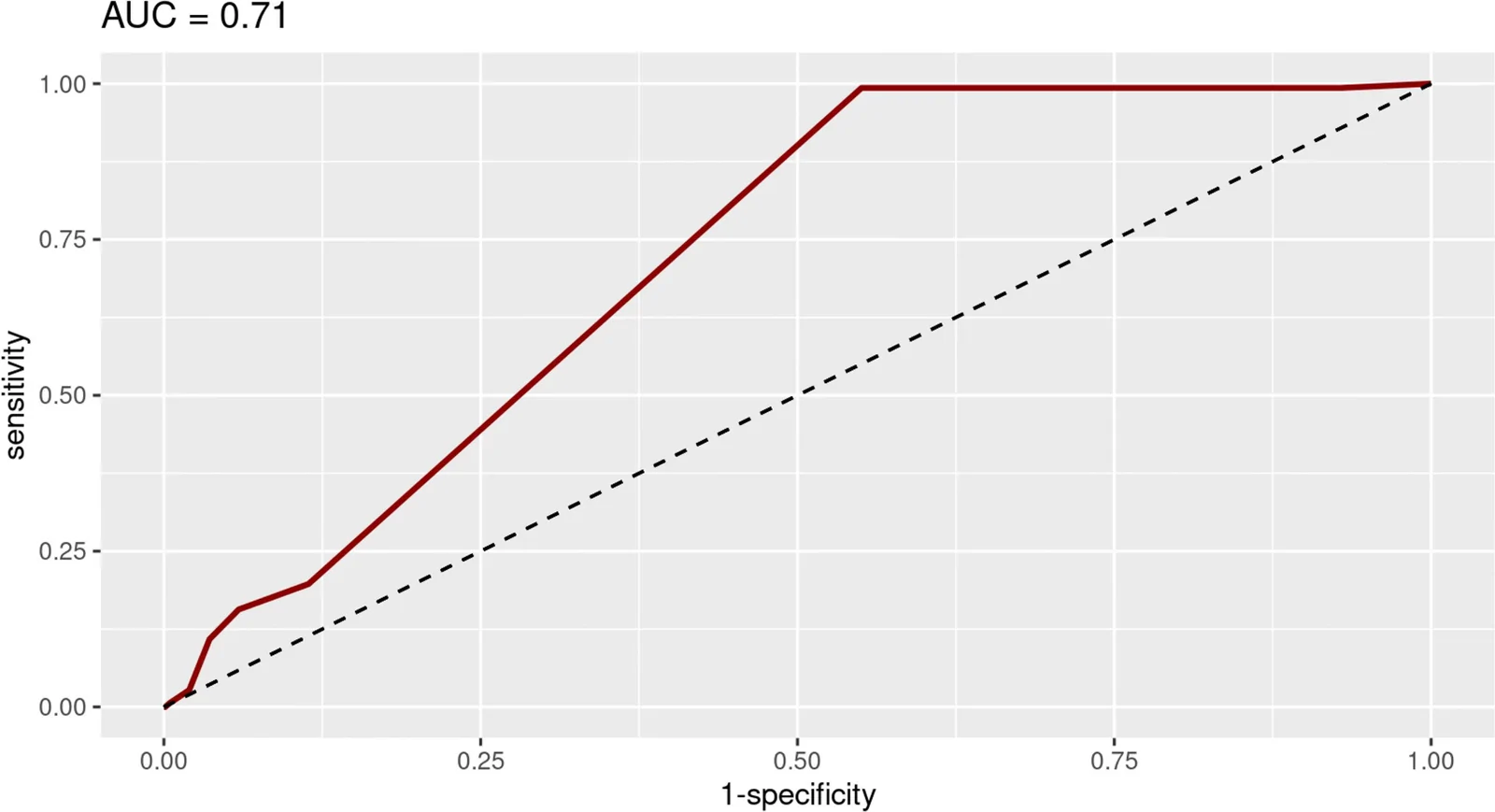
ROC curve for external validation of LIV-HITS

Using the referral outcomes shown in Table 5, the overall sensitivity of LIV-HITS was 99.3% (95% CI: 96.3%−100.0%) and specificity of 43.9% (95% CI: 41.3%−46.6%) (Table 6) (Fig. 2). The positive predictive value of the score was 15.7% (95% CI: 15.1%−16.3%) and negative predictive value was 99.8% (95% CI: 98.9%−100.0%). The diagnostic accuracy of the model was fair, with an AUC of 0.71, 95% CI 0.66–0.76 (Fig. 2).

**Table 6:** Diagnostic statistics of LIV-HITS

Sensitivity (95% CI)	Specificity (95% CI)	PPV (95% CI)	NPV (95% CI)
99.3% (96.3%−100.0%)	43.9% (41.3%−46.6%)	15.7% (15.1%−16.3%)	99.8% (98.9%−100.0%)

In the six-month data collection period, there were a total of 615 patients (39.8% of total referral volume) who were classed as true negatives by the scoring system as having a TBI that could potentially be managed through a non-emergency stream. Assuming similar referral volumes, this would equate to approximately 1200 less emergency mild TBI referrals being processed through the emergency on-call neurosurgery stream at the study unit.

## Discussion

To our knowledge, this is the first external validation of a scoring system, LIV-HITS, which can be used to stratify mild TBI referrals for emergency neurosurgical consideration. This study reproduces the internal validation’s demonstration of sensitivities over 99%, confirming that the LIV-HITS can accurately exclude a mild TBI that does not require urgent neurosurgical management, thus being a safe ‘rule-out’ tool for mild TBI requiring neurosurgical intervention. Where there was discrepancy between the LIV-HITS and actual management was an admission due to nonclinical reasons alone, further evidencing the safety of this approach. This is reflective of the original study, where one of the two cases admitted with a score < 3 was due to lack of beds in the referring centre, rather than as a surgical necessity [9]. It is important to note that LIV-HITS was not in use during the data collection period for the external validation cohort, and therefore could not bias decision-making when assessing its performance metrics. The fact that 615 referrals were correctly identified as potentially being managed successfully through a non-emergency management stream signifies a reduction of around 1200 mild TBI referrals to the emergency on-call service per year if the score was implemented when accounting for total referral volume, with all of these having a rapid decision for guidance and management, effectively being triaged to either other avenues or having rapid advice and guidance.

The high sensitivity (99.3%) and high negative predictive value (99.8%) of the score are of great importance to its diagnostic utility. In this cohort, the transfer rate (9.5%) was low, which reflects the national trend, and international cohorts that indicate a low rate of mild TBI requiring emergency surgery [30]. Mild TBI has significant heterogeneity in clinical and radiological presentation, often requiring different management strategies, ranging from observation and serial imaging to neurosurgical procedures [33]. Though at times mild TBI requires a specialist opinion, this is due to the specific indications required to rationalise a neurosurgical procedure or admit a patient to a tertiary neurosurgery centre for observation ± medical management [37, 38], such as the baseline status of the patient and their ability to tolerate an operation. In the East of England region, a quarter of residents are projected to be aged over 65 years by 2043 [32]. In this population, a neurosurgical operation may often not be indicated due to factors such as frailty, multiple co-morbidities, antiplatelet or anticoagulant use, or patient refusal [3, 25]. We also acknowledge that GCS can be reduced for entirely non-surgical reasons (e.g. intoxication, iatrogenic) and it is perhaps a weakness of the referral system that these patients, who may have no surgical target, may be more readily discussed than a patient with a potentially expansile contusion taking blood thinning medication who is currently GCS 15. Regardless, the ability to streamline patients to the appropriate management stream, including additional support following the acute phase of a mild TBI, may be a benefit of the score.

In this cohort, the specificity of the LIV-HITS was lower (43.9% vs 51.9%) than in the original study. This can potentially be explained by the high volume of chronic subdural haematomas (score of at least 3), that are again seen in the more elderly population of the East of England region [1, 28]. The large volume of subdural haematomas generally is also reflected by the fact that the most common score for both the accepted and non-accepted groups in our cohort was a ‘3’. A related point is that age as a variable was not factored into the development or validation of the LIV-HITS, as we felt that some of the most common associated pathologies (for example, chronic subdural haematomas in the elderly population vs contusions in the younger) would dichotomise and bias the dataset.

Taken together, our findings point towards its use as a post-CT ‘rule out’ triage tool for mild TBI, to stratify patients to the most appropriate referral and advice stream- whether this be acute neurosurgical advice, guidance, or transfer, or other services, such as post-concussion follow up, or outpatient clinic review. Importantly, the HITS score threshold does not represent an absolute distinction between specific injury combinations, but rather a cumulative indicator of radiological injury burden. These distinctions are important as ultimately, management decisions rely on clinical judgement of current neurological status as well as imaging to estimate risks and benefits of neurosurgical input [37, 38]. While many scoring systems exist for mild TBI, this is the first one to help make a disposition decision, after clinical and radiological evidence has been obtained. Tourigny et al. retrospectively evaluated the updated Brain Injury Guideline (uBIG) rules developed by Martin et al. in 2018 for the prediction of radiological deterioration and necessity of neurosurgical intervention [15, 20]. In validation of uBIG, a cohort of 477 patients with mild TBI, 8.4% received neurosurgical intervention, which is a similar figure to our study [36]. When assessing need for transfer using uBIG, their sample had a sensitivity of 100% (95%CI: 93.2%−100%) and a specificity of 9.4% (95%CI: 6.8%−12.6%). BIG utilises medication profile, in addition to including moderate and severe TBI and CT scan results. HITS is specifically for mild TBI, and only utilises CT results. Other decision making tools such as the modified Brain Injury Guideline (mBIG) rules and Quebec Brain Injury Categories (QueBIC) have been validated and shown to be clinically safe and effective [17, 35]. However they, as with the uBIG rules, are not prediction models which purely make a clinical disposition decision (in our case whether a patient is transferred to a specialist unit) as a primary endpoint. While this may not be translatable beyond the United Kingdom, HITS could be used as a proxy tool to highlight mild TBI that may require emergency neurosurgical intervention.

It is important to note that the external validation of the LIV-HITS presented here has its sensitivity and specificity calculated after a CT scan has been obtained, rather than as a decision-making tool for having a scan in the first place. As such, this study's specificity (43.9%) can also be interpreted as a potential percentage reduction out of all referrals rejected for transfer. LIV-HITS is therefore complementary to the currently most commonly used scoring systems in mild TBI; the clinical decision rules (CDRs) to aid decision for CT including the CT in head injury patients (CHIP) rule, New Orleans criteria (NOC), Canadian CT head rule (CCHR), and the NICE guidelines for head injury [8, 29, 31, 36]. The data presented here is supportive of future clinical pathways including a CDR before CT and LIV-HITS after.

Although LIV-HITS is an intuitive scoring system to use, in clinical practice it may be subject to a degree of inter-observer variability and is limited to national policies and infrastructure [4, 18]. The development of convolutional neural networks capable of multi-segment lesion classification suggests that in the future, there may be scope for machine learning (ML) screening tools to ‘rule in’ (or out) a surgically significant TBI with a high degree of accuracy [4, 18]. The fact that LIV-HITS has a negative predictive value of > 99% demonstrates that it is a useful ‘rule out’ score, however a ML tool with a high externally validated positive predictive value would be of great potential use to clinicians. A systematic review by Hibi et al. [11] in 2022 identified that many components of the LIV-HITS, such as intracranial haemorrhage classification, haematoma volume and haematoma location, have been individually studied and automated in existing ML studies [5, 11]. This demonstrates potential for the future development of AI-based mild TBI decision making tools, which may be able to highlight urgent referrals with precision.

This study has relevant implications for real life clinical practice. Further triaging up to 40% of mild TBI referrals, as opposed to being directed towards the on-call neurosurgical service would allow the on-call surgeon to spend more time on other urgent or emergent cases that require intensive decision making [21]. Depending on the set-up of the referral process used, it may also have the potential to alleviate financial costs on the units involved (potentially allowing for a redistribution to other non-neurosurgical services for patients with mild TBI). Moreover, the LIV-HITS is a straightforward and intuitive score to use, which in most cases can be instantly calculated once a CT scan is reported in a referring unit. It also provides benefit from the side of the referrer, in that it provides an objective tool that can increase their confidence of ruling out a significant injury, and that there is no delay in waiting for a neurosurgical reply once the score is calculated, improving hospital bed flow and resources. Ease of use and translatability to real life clinical practice are very important considerations when considering the significance of the development and validation of clinical prediction models [7]. However, we also acknowledge that the score may not be as readily utilised in resource limited healthcare systems, where the focus of the neurosurgical referral is to decide whether imaging is done at all, after a thorough history and examination. This may be a limitation of the score's wider clinical usability, although it is designed for use in the UK healthcare model, where neurosurgical referrals are usually made via online or telephone services to specialist centres. Instead, we recommend international units utilise the score as a discriminatory ‘rule out’ tool for highlighting mild TBI that may require surgical management, to help aid clinical decision making, without focussing on referral volume and patterns.

Importantly, potential reductions in referrals should be interpreted with caution. It should not be interpreted as restricting access to communication channels which, while may be beneficial, may not be possible within local policy and national governance frameworks. LIV-HITS is aimed to support consistent identification of mild TBI patients who may be safely managed at local non-neurosurgical centres. Specialist input would remain available, rather instead preserved for when clinical concerns remain or fall beyond the capture of the score. This thereby helps to streamline workflows without closing appropriate referral channels. Discussion of any CT abnormality with a neurosurgeon has its advantages. From the perspective of the referrer, specialist advice provides reassurance in clinically uncertain cases, and may reduce the risk of under-detecting patients who subsequently deteriorate. Current NICE guidance (NG232) supports discussion when imaging shows a new and surgically significant abnormality, as agreed locally between neurosurgical centres and referring institutions [24]. Greater availability of neurosurgical discussion would therefore facilitate this. However, this approach is not without costs. Universal discussion of all CT abnormalities, irrespective of clinical relevance or severity, would likely increase referral volume and delay neurosurgical advice to patients at higher risk. For patients, it can contribute to longer hospital stays and can lead to an increase in unnecessary transfers. LIV-HITS may thus act as a pragmatic decision support tool to help consistently identify patients who may be managed safely locally with expedited advice.

It should be noted that a whole systems, integrated approach is needed to meaningfully improve the current TBI care pathway. In some systems, hospital bed availability can be either readily available or an infrastructural challenge. We struggled to incorporate an objective measure of this into our score. While bed planning is a key consideration, so too are pressures across the wider hospital system, including in emergency departments. Patient access to specialist TBI services, such as neuropsychology and rehabilitation, is crucial to improve long term outcomes, and the HITS score would ideally support implementation of a wider, more co-ordinated model that matches patients with their appropriate team whilst also accessing non-surgical TBI expertise and allowing access to specific neuropsychological and neuropsychiatric support despite not being admitted to a tertiary neurosurgery centre.

### Study limitations

A limitation of this study is its retrospective nature within a single centre. It is thus potentially subject to a bias with the clinical decision, which was made prospectively, but with scans scored retrospectively [2]. Additionally, although a CT scan was a part of all referrals included in the study, in some cases the on-call neurosurgeon was contacted for advice regarding restarting anticoagulation, for instance, rather than a decision on surgery or transfer of the patient. However, they were still included by the investigators if a CT scan was present that could be evaluated by the HIT score.

Additionally, it is pertinent to mention that using transfer as a standardised outcome may create issues with generalisability. Different patients would have had different management plans on admission to our unit, such as whether they were initially transferred to the ward or neuro-intensive care unit, or whether they had a neurosurgical intervention at all. The study also did not assess mortality as an outcome, which would provide a wider evaluation of the transfer service, perceived safety of transfer, and subsequent neurosurgical management (if any).

Another limitation relates to the potential circularity surrounding ‘surgically significant’. As reflected by NICE guidance (NG232), the concept inherently remains shaped by regional practice [24]. LIV-HITS may thus in part reflect UK practice with respect to what counts as ‘significant’ CT abnormalities, rather than necessarily represent a novel discriminator. While external validation is encouraging, both the East of England and Liverpool cohorts still remain within the UK context, thereby limiting broader generalisability.

There were also certain cases where although a patient had a score of less than 3, a discussion with a neurosurgeon may have been appropriate. These were mild TBI cases with a previous neurosurgical history such as a presence of a CSF shunt or history of seizures, despite no ‘surgically significant’ pathology on imaging. Patients may also present with other significant neurosurgical pathology that merits discussion with a neurosurgeon, despite not having a ‘surgically significant’ CT head scan; for example incidental lesions including meningioma or arachnoid cysts. Referrers may also be contacting for general advice, such as when to restart anticoagulation therapy. This highlights the need for using the score to stratify advice specifically about TBI management, whilst applying clinical judgement for the overall patient context.

The presentation of a head injury is also complex and multi-faceted. Therefore the score, which is solely based on CT scan result alone, may oversimplify or downplay these factors. There is a need to ensure that referrers have access to neurosurgical consultation regardless of HITS score. It is also possible that LIV-HITS represents UK referral practice, rather than a novel mild TBI discriminatory tool. We suggest the score is trialled in other centres internationally to establish if a similar diagnostic accuracy is obtained.

## Conclusion

The Liverpool Head Injury Tomography score is a potentially useful tool in clinical practice to confidently rule out need for emergency neurosurgical advice and management in a mild TBI population who have had a CT head. It has a high diagnostic power with sensitivities of over 99.0% on internal and external validation. Its application into clinical practice may aid development and implementation of clinical pathways to improve care of patients after mild TBI and their outcomes.

## Data Availability

Data is available on reasonable request by contacting the corresponding author of the study.
